# Information spread behavior of clubhouse: A value-attitude-behavior model perspective

**DOI:** 10.1016/j.heliyon.2024.e39377

**Published:** 2024-10-16

**Authors:** Yu-Feng Wu, Yu-Tai Wu, Jian-Hong Ye, Jhen-Ni Ye

**Affiliations:** a Office of Physical Education, Ming Chi University of Technology, New Taipei City, Taiwan; b Office of Physical Education, Soochow University, Taipei, Taiwan; c Faculty of Education, Beijing Normal University, Beijing, China; d Graduate Institute of Technological & Vocational Education, National Taipei University of Technology, Taipei, Taiwan

**Keywords:** Information spread behavior, Internet meme (网络迷因), Social value, Value-attitude-behavior model, Voice social media, Immediate response syndrome

## Abstract

The prevalence of social media has significantly facilitated the spread and diffusion of online memes. The Clubhouse voice community application, which gained popularity rapidly during the COVID-19 pandemic and became widely used, can be regarded as a form of internet meme. However, the underlying reasons for this phenomenon have been scarcely explored. In this context, the purpose of this study is to understand the dissemination mechanisms of the Clubhouse voice community. Specifically, this research employs the value-attitude-behavior model to investigate the relationships between social value, attitude, immediate response syndrome, and information spread behavior on Clubhouse. To achieve this objective, the snowball sampling technique was used to recruit Clubhouse users to complete a questionnaire. The collected data were assessed for reliability and validity before being analyzed using structural equation modeling for confirmatory factor analysis and model verification. The results indicated that the social value of joining Clubhouse is positively associated with participants’ attitudes toward participation. Additionally, attitudes toward participation were found to be positively associated with both immediate response syndrome and information spread behavior, thus showing a positive association between immediate response syndrome and information spread behavior. This study reveals the pathways of internet meme dissemination, offering insights into the factors driving the formation of internet memes.

## 1 Introduction

Powerful smartphones and other mobile devices have given rise to numerous social media applications (apps) and have become channels of communication for many people. As a result, social media now permeates the daily lives of online users [1]. Beyond everyday communication, social media serves as an important medium for sharing information about leisure and family recreational activities [2]. With mobile phone use being deeply integrated into everyone's daily routines, many studies have explored the social and psychological effects of their usage. However, despite the distinctive features of mobile social media and the notable growth of mobile devices, few studies have specifically examined the impact of mobile social media on interpersonal relationships, especially considering that mobile phones offer unique functions compared to traditional phones [3]. The rapid development of social media as a digital platform has revolutionized how people connect and share information. This evolution has enabled novel methods of personalized social interaction [4] and has given rise to the creation of internet memes, which spread rapidly by internet users across many social media platforms.

The concept of the internet meme involves the rapid transmission of influence, preferences, and behaviors within a social network [5]. The prevalence of social media has significantly contributed to the spread and proliferation of online memes [6]. A notable example is the Clubhouse voice social app, which rapidly gained popularity and widespread use [7]. It quickly emerged as one of the most important social media networks, with its user base increasing fivefold between January and February of 2021, reaching 10 million active users per week [8]. While scholars have long examined how social media platforms influence user communication and behavior [9] and while research on internet memes—particularly those popular among younger users—has flourished, the factors behind the success and diffusion of memes remain unclear [10]. Therefore, this study seeks to understand the information spread behavior (ISB) of Clubhouse-based internet memes.

Previous research has predominantly focused on information spreading dynamics on traditional social media platforms, such as Facebook and Twitter, leaving a notable gap in understanding these dynamics within voice-based social networks like Clubhouse [11]. To address this gap, this study investigates the following research question: How do social value, attitude, and immediate response syndrome contribute to ISB on Clubhouse? By applying the value-attitude-behavior (VAB) model to the novel context of voice-based social networking [12,13], this research adds additional value to previous literature. With the focus on a relatively new and underexplored platform like Clubhouse, this study helps develop a deeper understanding of information spread in digital environments. Therefore, this study used the value-attitude-behavior model to investigate the relationship between social value, attitude, immediate response syndrome, and ISB on Clubhouse.

## 2 Literature review

In the 21st century, digital culture has significantly influenced people's leisure patterns [14,15], with social media becoming interconnected with daily leisure experiences [2]. As a result, it has become increasingly important to understand how these new digital practices have evolved into common leisure behaviors [15]. To understand the social psychology of this phenomenon, the VAB model has been widely used. This model consists of three factors: values, attitudes, and behaviors. Values represent the ideals and fundamental principles that guide people's actions [16,17] and help trace the causal pathway from abstract cognition (values) to moderate cognition (attitudes) to specific behavior [18]. This approach differs from earlier models that treated perceived value as a mediating or dependent variable; instead, it highlights the importance of value itself. Therefore, this study aims to develop a model for understanding ISB on social media using the VAB theory and to validate this model through structural equation modeling.

### 2.1 The Clubhouse app

**Fig. 1:**
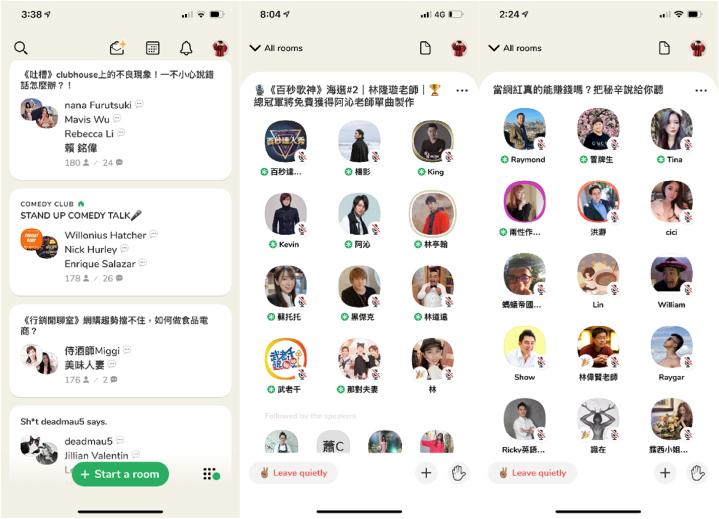
The Clubhouse User Interface Note: Screenshot of the Clubhouse application interface.

Founded in February of 2020 in Silicon Valley, Alpha Exploration launched Clubhouse, an invitation-only community app for iOS users. Upon its release, Clubhouse provided a space where members could chat in an intimate environment [19]. Users can either create chat rooms or directly enter rooms that interest them, where they can share topics and stories, engage in group conversations, and exchange ideas. Within these rooms, participants can take on one of three identities: moderator, listener, or speaker. Those with microphones are able to chat with each other through voice discussion, while others can either listen or “raise their hands” to request to speak at any time [20]. Additionally, users have the option to use pseudonyms to conceal their identities [21], as shown in Fig. 1.

Clubhouse describes itself as “a platform for connecting with friends and other interesting people from around the world” [22]. The platform attracts a diverse range of users, including musicians, scientists, creators, athletes, comedians, entrepreneurs, stock traders, nonprofit leaders, writers, artists, real estate agents, sports fans, and more. This diversity allows users to engage in enjoyable voice conversations with people from different backgrounds and life experiences [23].

### 2.2 Social value

Socialization is particularly derived from real and local experiences, new and enjoyable encounters, conversations, and cultural insights [24]. It can be described as the pleasure and satisfaction that individuals receive from participating in interpersonal interactions [25]. Social value is reflected through friendships, social support, and intimacy, which are cultivated through one's participation and interaction with other members [26]. Therefore, social value plays an important role in determining individuals' usage behavior.

Value is a key concept in the VAB model [27] and is defined as the result of multiple interactions between different participants [28]. For instance, a study stated that trust and perceived value drive health-related information behavior, aligning with the concept of social value, as users are more likely to engage in spreading information when they perceive the content as valuable [29]. Social websites and apps serve as online communities where members can create profiles, disseminate information, and interact with others [30]. These platforms help individuals build interpersonal relationships by reducing the social distance within the social media medium [31]. In addition, social media platforms facilitate connections between users from different backgrounds, thereby creating a diverse social structure [32]. Moreover, their dynamic, interconnected, egalitarian, and interactive nature has been described [33]. This demonstrates that positive social interactions can lead to a positive perception of social value among users. In this study, social value refers to the perceived value of social interactions within Clubhouse and is used to investigate how participants perceive their social value from joining the platform.

### 2.3 Attitude toward participation

Another key component of the VAB framework is attitude [27], which Ajzen defined as a person's evaluation of behavior as being either good or bad [34]. Attitude encompasses beliefs about a behavior formed after evaluating its expected outcomes [35] and is also associated with specific characteristics of a product or service that affect an individual's positive or negative feelings about participating in a particular event [36]. Social networking sites are widely considered as media for individuals to develop either favorable or unfavorable attitudes, with attitudes towards participation being shaped by such factors as perceived information interactivity [37]. In this study, attitude toward participation in Clubhouse refers to users' perceptions of their attitudes towards the interactivity of presentations by Clubhouse speakers.

### 2.4 Immediate response syndrome

Highly synchronized media prompt people to engage in immediate response behavior [38], characterized by rapid responses in online settings [39]. The term “immediate response syndrome” explains the behavioral tendency of users to respond immediately upon receiving a message because they are in a state of temporary unease until they respond [40]. Notifications create pressure to respond promptly, as delayed responses may undermine the sender's expectations. The fear of missing out can exacerbate immediate response syndrome, as users may feel compelled to share information quickly to avoid missing out [41]. Consequently, people often try to minimize response times after receiving notifications. Additionally, trust can influence users' attitudes toward information and their likelihood of sharing it. This connection is particularly relevant in the context of immediate response syndrome, where users may rapidly share and react to data without verifying their accuracy [42]. In other words, users feel compelled to respond immediately after receiving a message and tend to increase the frequency of their responses. In this study, immediate response syndrome refers to how quickly users react to event reminder notifications from Clubhouse (e.g., room openings, friends' online status, and event notification).

### 2.5 Information spread behavior

Recent advancements in Information and Communications Technology (ICT), alongside developments in the media and entertainment industries, have led to the emergence of new service platforms [43]. For instance, modern social media platforms enable information dissemination through user actions like retweeting tweets or rating content. On these platforms, users are often influenced by others, forming opinions and taking actions that contribute to the process of information dissemination [44]. Guadagno et al. suggested that people can spread information rapidly online, which exacerbates its contagiousness [45]. In a study by Allsop et al. [46], 59 % of participants reported frequently or very frequently forwarding internet materials to colleagues, peers, family, or friends. In the digital age, phenomena, such as viral events or trending content, capture the attention of many internet users, who then follow or spread this information across social networks. Internet memes, in particular, have become a defining feature of the 21st-century digital culture and an important component of social interactions [47]. In this study, ISB refers to the behavior of Clubhouse users in communicating and sharing Clubhouse-related information, including conversation content and topics.

## 3 Research model and hypotheses

### 3.1 Research model

**Fig. 2:**
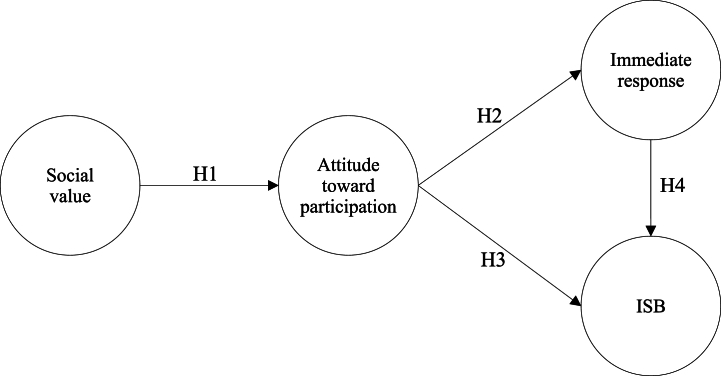
Research model.

Internet memes exemplify the trend in today's participatory culture, where digital content is widely spread, copied, altered, and then disseminated across online communities [48]. People often engage with internet memes to spark social interactions, driving the constant iteration and emergence of new memes online. In social psychology, the VAB model has been widely used to understand behavior [49] and can be applied to the ISB of internet memes. According to the VAB model, the influence of values on specific behaviors is mediated by attitudes, suggesting that influence progresses from values to attitudes and then to specific behaviors [18,50]. In other words, when individuals assess the value of an activity, it shapes their preferences and behavioral outcomes. Moreover, the hierarchy of values, attitudes, and behaviors appears to hold across different countries and cultures [51]. Based on this perspective, this study proposes a model to explore the relationship between social value, attitude toward participation, immediate response syndrome, and ISB, as illustrated in Fig. 2.

### 3.2 Social value and attitude toward participation

Value serves an important prerequisite for attitude [52] and acts as a guiding principle for directing individual behavior. It provides a standard to evaluate people's behavior, including other behavioral determinants, such as beliefs and attitudes [53,54]. Past research has consistently demonstrated that the perceived value of new technologies helps customers develop favorable attitudes [55]. For example, research has found that cognitive processes and algorithmic decisions influence user engagement and the adoption of technological platforms. In the context of Clubhouse, similar cognitive mechanisms may be at work, where users' perceived social value and attitudes are shaped not only by social exchanges but also by algorithmic indicators embedded in the platform [56]. Due to the widespread use of mobile phones and the internet, perceived value plays a significant role in shaping attitudes toward technology usage [57]. Based on this understanding, it can be inferred that when users perceive strong social value in Clubhouse, they are more likely to have a more positive attitude toward participating in the platform. Consequently, this study proposes the following hypothesis regarding the relationship between social value and attitude toward participation.

H1 Social value is positively related to attitude toward participation.

### 3.3 Attitude toward participation and immediate response syndrome

Users’ attitudes toward participation in social media influence their subsequent usage behavior [37]. Research has shown that positive attitudes toward social media use often result in more frequent use of these platforms [58]. Specifically, studies have indicated that extended time spent on the internet increased the likelihood of responding immediately to messages [59]. In other words, higher internet usage correlates with more frequent and immediate response and communication [60]. Previous studies have further supported this, suggesting that greater internet usage is associated with higher trends of immediate response syndrome [40]. Therefore, it can be inferred that users with a more positive attitude toward participating in Clubhouse are more likely to respond quickly to new Clubhouse messages. Based on this reasoning, the following hypothesis regarding the relationship between attitude toward participation and immediate response syndrome is proposed.

H2 Attitude toward participation is positively related to immediate response syndrome.

### 3.4 Attitude toward participation and ISB

Attitude is based on the expectation of an outcome and is defined as an individual's favorable or unfavorable assessment of behavior, such as whether or not to perform a behavior well [61]. Various social psychological theories have assumed that attitude is often used as a volitional factor in shaping an individual's future behavior [62,63], and the literature on attitudes has suggested that behavioral performance will be influenced by positive or negative attitudes [64]. Therefore, attitudes are often considered to be an important factor in inducing behavior [65,66]. In addition, Abrahamse et al. and Muñoz et al. found a direct relationship between attitudes and behaviors [67,68]. Based on this reasoning, it can be inferred that when users have a positive attitude toward participating in Clubhouse, they will engage more frequently in Clubhouse-related ISB. Therefore, this study proposes the following hypothesis.

H3 Attitude toward participation is positively related to ISB.

### 3.5 Immediate response syndrome and ISB

When users have the tendency to tolerate delayed responses, they are unable to refrain from censoring social media [60], and in social media, receiving a message often leads to a desire to spread it [69]. In terms of internet memes, people tend to spread messages after they receive them, as memes are quickly copied and spread by internet users [70]. From the perspective of communication theory, the four main factors affecting communication are innovation, time, communication channel, and social system [71]. In summary, participants' ISB increases with a higher tendency toward immediate response syndrome. Therefore, this study proposes the following hypothesis.

H4 Immediate response syndrome is positively related to ISB.

## 4 Methodology

### 4.1 Research design and procedure

This study employed a cross-sectional validation design that utilized the snowball sampling method to recruit participants. A link to the survey was placed on Clubhouse's profile for access by ethnic Chinese users of Clubhouse. Participants completed the survey anonymously and were encouraged to forward the questionnaire link to friends. The questionnaires were also distributed within the Clubhouse user groups. The first page of the web questionnaire included a consent statement informing participants that their responses would remain completely anonymous, their privacy would be protected, and their data would be used solely for research purposes. Participation was voluntary, and all participants provided informed consent statements. Data collection occurred from February 15 to February 28, 2021, during a period of rapid growth and media focus on Clubhouse.

### 4.2 Participants

A total of 364 questionnaires were collected, with 58 invalid data removed, resulting in a valid sample size of 317 and a valid recall rate of 87.1 %. Among the respondents, 183 (57.7 %) were male and 134 (42.3 %) were female. In terms of usage, 272 (58.8 %) were former users and 45 (14.2 %) were non-users. Usage frequency was categorized as follows: 9 (2.8 %) used the platform once a week or less, 52 (16.4 %) used it two to three times per week, 85 (26.8 %) used it four to five times per week, 119 used it six to seven times per week (37.5 %), and 52 used it more than 1 time per day (16.4 %). Regarding duration, 67 participants (21.1 %) used it for less than 1 h each time, 149 (47 %) used it for 1–2 h each time, 81 (25.6 %) used it for 3–4 h each time, and 20 participants (6.3 %) used it for more than 5 h each time. The average age of participants was 26.71 years (*SD* = 6.22).

### 4.3 Measurements

The measurements used in this study were adapted or designed based on variable definitions and evaluated using a 5-point Likert scale, where 1 was defined as “Strongly Disagree,” 2 as “Disagree,” 3 as “Neutral,” 4 as “Agree,” and 5 as “Strongly Agree.” The initial questionnaire was reviewed by three psychometrics experts for alignment with variable content, scale completeness, and clarity of wording.

#### 4.3.1 Social value

Social value encompasses friendships, social support, and intimate relationships gained through participation and interaction with other members [26]. This study adapted Ye et al.'s social value construct to measure participants' perceived social value when using Clubhouse [72]. For example, “I met more friends from different cultural backgrounds after using Clubhouse.”

#### 4.3.2 Attitude toward participation

Attitude toward participation refers to users’ favorable or unfavorable attitudes toward a social networking site [37]. Based on this concept, a six-item questionnaire was designed to measure participants' attitudes toward using Clubhouse. For example, “if someone recommended a room on Clubhouse, I would be eager to participate.”

#### 4.3.3 Immediate response syndrome

Immediate response syndrome is characterized by users’ need to respond immediately upon receiving a message, resulting in temporary difficulty with disconnecting from their phones [40]. Based on this concept, a six-item questionnaire was designed to measure participants' immediate response syndrome related to the use of Clubhouse. For example, “when I receive a new event notification on Clubhouse, I immediately check it.”

#### 4.3.4 Immediate response syndrome

ISB is driven by user actions, such as retweeting or rating content [44]. Based on this concept, a questionnaire was designed to measure participants' behavior related to spreading information about Clubhouse. For example, “I participate in Clubhouse conversations and then repost them in other online communities.”

### 4.4 Statistical methods

This study used confirmatory factor analysis (CFA) for testing. First, a first-order CFA was conducted to confirm that the measurement dimensions had acceptable internal and external validity. Next, reliability and validity of these dimensions were tested to ensure that they met the necessary requirements. Then, model fit analysis was performed. Finally, after meeting all numerical criteria recommended by statisticians, the research model and hypotheses were tested.

## 5 Results and discussion

### 5.1 First-order confirmatory factor analysis

Following the required threshold values of the model and residuals, the χ2/df ratio must be lower than 5, the RMSEA must be lower than 0.10, the GFI and AGFI values must be higher than 0.80, and the factor loading (FL) value of the question should be larger than 0.50 [73,74], as shown in Table 1. The results of the deletion in this study were as follows: social value was reduced from seven items to five; attitude toward participation from six to five; immediate response syndrome from six to five; and ISB from six to five.

**Table 1:** First-order confirmatory factor analysis.

Index	Threshold	Social value	Attitude toward participation	Immediate response syndrome	ISB
χ^2^	–	9.7	19.9	13.5	19
df	–	5	5	5	5
χ^2^/df	<5	1.94	3.98	2.70	3.80
RMSEA	<0.1	0.05	0.09	0.07	0.09
GFI	>0.80	0.99	0.98	0.98	0.98
AGFI	>0.80	0.97	0.97	0.95	0.93

### 5.2 Reliability and validity analysis

Hair et al. suggested that Cronbach's α and composite reliability (CR) values greater than 0.70 indicate acceptable internal consistency reliability and compositional reliability [73]. In this study, both the α and CR values were greater than 0.84. McClure et al. suggested that FL and average variance extracted (AVE) values greater than 0.50 indicate convergent validity [75]. The FL values in this study ranged from 0.69 to 0.80, as shown in Table 2.

**Table 2:** Reliability and validity analysis.

Index	Threshold	Social value	Attitude toward participation	Immediate response syndrome	ISB
*M*	–	3.57	3.48	3.48	3.52
*SD*	–	0.56	0.58	0.59	0.57
α	<0.70	0.88	0.84	0.86	0.84
*FL*	<0.50	0.69–0.80	0.69–0.74	0.70–0.79	0.68–0.75
CR	>0.70	0.88	0.84	0.86	0.84
AVE	>0.50	0.59	0.54	0.55	0.51

Discriminant validity refers to the degree of differentiation between constructs [76]. To assess this, the square root of the AVE for each construct should be compared to the absolute value of correlation coefficients between constructs. The results of the analysis showed that the square root of the AVE for each construct was higher than the corresponding correlation coefficient values, indicating that the construct had discriminant validity, as shown in Table 3.

**Table 3:** Discriminant validity analysis.

Constructs	1	2	3	4
1. Social value	(0.77)			
2. Attitude toward participation	0.42	(0.73)		
3. Immediate response syndrome	0.48	0.47	(0.74)	
4. ISB	0.57	0.60	0.66	(0.71)

### 5.3 Model fit analysis

AMOS 20 was used to conduct the fit analysis and final validation of the study model. The suggested fit indices for evaluating model fit include a χ2/df less than 5 [73]; RMSEA less than 0.10; GFI, AGFI, NFI, NNFI, CFI, IFI, and RFI greater than 0.80 [77]; and PNFI and PGFI greater than 0.50 [73]. The fit indices for this study were as follows: χ2 = 340.9, df = 166, χ2/df = 2.05, RMSEA = 0.06, GFI = 0.90, AGFI = 0.88, NFI = 0.90, NNFI = 0.94, CFI = 0.94, IFI = 0.94, RFI = 0.88, PNFI = 0.78, PGFI = 0.71. These values were all in accordance with the recommended standards.

### 5.4 Path analysis

**Fig. 3:**
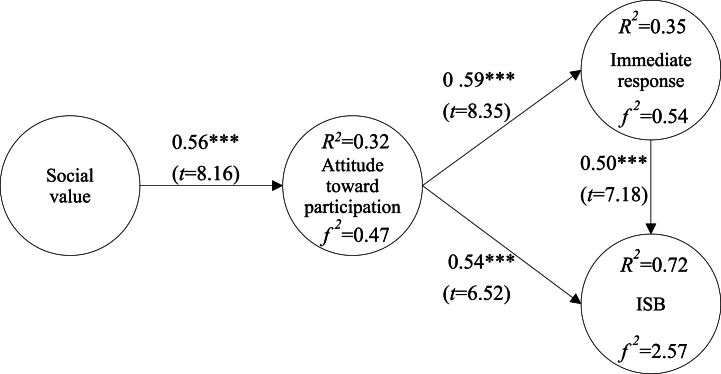
Verification of the research model.

The results of the model validation showed that social value was positively associated with attitude toward participation (*β* = 0.56 ∗∗∗; *t* = 8.16); attitude toward participation was positively associated with immediate response syndrome (*β* = 0.59∗∗∗; *t* = 8.35); attitude toward participation was positively associated with ISB (*β* = 0.54∗∗∗; *t* = 6.52); and immediate response syndrome was positively associated with ISB (*β* = 0.50∗∗∗; *t* = 7.18). The explanatory power of social value on attitude toward participation was 32 % and *f2* was 0.47; the explanatory power of attitude toward participation on immediate response syndrome was 35 % and *f2* was 0.54; and the explanatory power of attitude toward participation and immediate response syndrome on ISB was 72 % and *f2* was 2.57, as shown in Fig. 3.

## 6 Discussion

### 6.1 Social value was positively related to attitude toward participation

Chung suggested that value is an important prerequisite for attitude generation, meaning that value affects the perceived outcome of attitude [52]. Kautish and Stegalso suggested that value can be used to examine people's attitudes [53,54], and Kim et al. (2021) found that past studies have consistently demonstrated the value of new technologies [55]. Perceptions of the value of new technologies contribute to the development of positive attitudes among users. Hsu et al. also confirmed the significant role of value in understanding attitudes towards the use of technology [57]. The results of this study confirmed that social value positively impacts attitude toward participation, which aligns with previous studies and suggests that a higher perceived social value of Clubhouse promotes a higher participating attitude toward Clubhouse.

### 6.2 Attitude toward participation is positively related to immediate response syndrome

Bailey et al. demonstrated that users' attitudes toward participation in social media influence their subsequent usage behavior [37]. Wang et al. confirmed that positive attitudes toward social media lead to more frequent use [58]. In addition, Kanoh looked at previous studies and suggested that a more frequent use of the internet leads to more pronounced immediate response syndrome [40]. Furthermore, Aoki suggested that a more frequent use of technology leads to more frequent immediate responses and communications by the user [60]. Kanoh and Chou also suggested this, finding that the longer individuals use the internet, the more likely they are to respond immediately to messages [59]. The results of this study confirmed that a positive attitude toward participation has a negative effect on immediate response syndrome, which is in line with previous studies. This suggests that the more positive attitudes users have toward using Clubhouse, the faster they will respond to incoming Clubhouse messages.

### 6.3 Attitude toward participation is positively related to ISB

Fishbein and Ajzen defined attitude as a person's favorable or unfavorable evaluation of a behavior, suggesting that attitude reflects the expected outcome of preforming that behavior [61]. Hurst et al. and Lee further proposed that attitude is an important factor in influencing behavior [65,66]. By contrast, Padilla-Meléndez et al. and Hwang argued that attitudes play a role in shaping an individual's future behavior across various social psychological theories [62,63]. Ajitha and Sivakumar also indicated that behavioral performance is affected by positive or negative attitudes [64]. Studies by Abrahamse et al. and Muñoz et al. have supported the notion that attitudes have both direct and indirect effects on behavior [67,68]. The results of this study confirmed that a positive attitude toward participation in Clubhouse has a positive effect on ISB, aligning with the results of previous studies. This suggests that users with more positive attitudes toward Clubhouse are more likely to engage in and propagate Clubhouse content more frequently.

### 6.4 Immediate response syndrome is positively related to ISB

Aoki et al. highlighted that individuals who have strong experiences of immediate response syndrome are unable to stop checking social media [60], which aligns with Lee et al.’s findings [71]. Aruguete and Calvo observed that message spreading on social media often follows the receipt of a message [69]. In addition, Plester and Inkson found that internet memes proliferate due to rapid copying and spreading by users [70]. The results of this study confirmed that immediate response syndrome positively affects ISB, consistent with previous literature. This suggests that users who quickly respond to Clubhouse messages are more likely to increase their ISB regarding Clubhouse content. Additionally, the study's verification of hypothesized paths indicated that process variables can influence behavioral outcomes.

### 6.5 General discussion

In 2021, during the peak of the global COVID-19 pandemic, there was a significant surge in the use of internet-related social software. In this unique context, Clubhouse gained rapid global popularity, establishing a vast virtual social network. When Clubhouse was initially launched, it provided a space where members could chat in an intimate environment [19]. The app's diverse user base, spanning various industries and countries, led to the creation of chat rooms on a wide array of topics. Within these rooms, moderators and speakers could present opinions from various professional fields and perspectives, while audience members were able to join in on the conversations, providing users with significant social value.

Moreover, Clubhouse's feature of not saving chat content heightened users' fear of missing out on information, making them more susceptible to immediate response syndrome. This usage mechanism also facilitated the spread of internet memes, which outlined the VAB model's stance that values serve as the foundation for attitude formation, which subsequently influence behavior. These findings emphasized the widely accepted scientific theories regarding the importance and relevance of perceived value in describing human behavior [78].

### 6.6 Limitations and future studies

This study has several limitations. First, it was conducted exclusively with Chinese respondents, which may limit the generalizability of the findings due to potential cultural differences. Future studies should explore ISB across different ethnic groups and cultural backgrounds, as well as conduct comparative studies that consider these factors. Additionally, according to situational expectancy-value theory, perceived value can vary with different contextual backgrounds. Therefore, incorporating contextual factors into the research model could offer a more nuanced explanation of the subtle variables in different situations and provide a more effective understanding of the research results.

Second, this study primarily utilized the VAB model to explore users' ISB and did not account for other influencing factors. Subsequent studies may consider adding other theories to identify the factors affecting users’ ISB. Furthermore, the mechanisms behind meme creation remain largely unexplored. It may be beneficial to consider implementing other research methods and perspectives to explore this further, allowing for a better understanding of the processes that shape internet culture.

Third, immediate response syndrome is an important process variable influencing users' behavioral habits on social media. Future research should incorporate immediate response syndrome, especially in studies focused on inappropriate usage behaviors, to provide a more comprehensive understanding of the positive and negative effects of immediate response syndrome.

Fourth, emotional value has gained prominence in recent years, referring to elements within products, services, or interactions that evoke emotional resonance and experiences, thereby meeting people's emotional needs. This type of value transcends the practicality emphasized by traditional products, focusing instead on addressing consumers' emotional needs and psychological satisfaction. Providing emotional value is crucial for establishing emotional connections between users of social media and reducing customer dissatisfaction due to functionality issues or product problems. Increased overall user satisfaction often leads to sustained engagement or continued use. It is suggested that future research incorporate emotional value to explore its role in different types of media usage, social interactions, or information dissemination.

Finally, when a digital media operator or content gains significant attention and followers through the spread of memes, they become a symbol of online culture. Their influence extends beyond the internet into different fields, and the 'internet celebrity effect' starts to take shape. As a result, the connection between memes and the internet celebrity effect becomes increasingly close. Therefore, in future research, the formation of the internet celebrity effect can also be analyzed from the perspective of memes, as well as some potential consequences that the internet celebrity effect may lead to.

## 7 Conclusions and implications

### 7.1 Conclusions

In this modern age, various aspects of life, including leisure activities, have become increasingly digitized. The concept of digital leisure culture now extends beyond the distinction of virtual and real. Scholars have long been interested in how social media platforms influence user communication and behavior. Internet memes, particularly popular among young social network users, are a notable example of this trend, yet the factors influencing their success and diffusion are still unclear. Therefore, this study contributes to the existing social networking service SNS literature by integrating internet memes with the VAB model and using empirical data from Chinese Clubhouse users worldwide to validate the ISB model.

The results of the study showed the following: 1. social value has a positive effect on attitude toward participation; 2. there is a positive effect of attitude toward participation on both immediate response syndrome and ISB; and 3. immediate response syndrome has a positive effect on ISB. In summary, this study revealed the role of social processes in information dissemination within the context of the VAB research model.

### 7.2 Implications

Past research has frequently treated perceived value as a mediating or outcome variable, often focusing on its direct impact. However, the VAB model suggests that values influence specific behaviors through attitudes toward those behaviors. Theoretically, influence should flow from values to attitudes and then to specific behaviors. This study confirmed that the VAB model effectively facilitates the occurrence of ISB on Clubhouse. In other words, when individuals perceive potential benefits, it shapes their perspectives and motivations. This finding enhanced our understanding of the role of perceived value as a critical antecedent variable, emphasizing its importance.

Another theoretical contribution of this study is the confirmation that immediate response syndrome serves as a crucial process variable in social software use, influencing the driving forces behind behavior. While immediate response syndrome aids in the rapid dissemination of information, it also has the potential to lead to increased time spent, higher frequency, or greater volume of interactions with social software as users strive to respond quickly to messages. The dual nature of immediate response syndrome highlights the need for caution and awareness to prevent overuse or addiction.

From a management perspective, the findings of this study offer valuable insights for social media developers and managers seeking to attract users to technology services. Social media, now a fundamental aspect of daily life, is frequently used for leisure or connecting with others. This shift presents opportunities for leisure peripheral products and companies to utilize social media sites to build marketplaces and promote their business. By doing so, this allows the leisure industry to create and exchange user-generated content.

## CRediT authorship contribution statement

**Yu-Feng Wu:** Writing – original draft, Methodology, Investigation, Funding acquisition, Conceptualization. **Yu-Tai Wu:** Writing – review & editing, Validation. **Jian-Hong Ye:** Writing – original draft, Project administration, Conceptualization. **Jhen-Ni Ye:** Writing – review & editing, Data curation.

## Informed consent

All participants provided informed consent statements.

## Availability of data and materials

The original contributions presented in the study are included in the article/supplementary material; further inquiries can be directed to the corresponding author.

## Research ethics

The research conducted in this study involves an online anonymous survey, with no participants undergoing any direct contact. Ethical review and approval was not required for the study on human participants in accordance with the local legislation and institutional requirements.

## Declaration of competing interest

The authors declare the following financial interests/personal relationships which may be considered as potential competing interests:Yu-Feng Wu reports financial support was provided by 10.13039/100020595National Science and Technology Council, Taiwan, R.O.C. Yu-Feng Wu reports was provided by Ming Chi University of Technology. Yu-Feng Wu reports a relationship with 10.13039/100020595National Science and Technology Council, Taiwan, R.O.C that includes: funding grants. Yu-Feng Wu reports a relationship with 10.13039/501100019786Ming Chi University of Technology that includes: employment and funding grants. Jian-Hong Ye is one of the associate editors of the Applied Psychology section of Heliyon, they declare that they have no known competing financial interests or personal relationships that could have appeared to influence the work reported in this paper. If there are other authors, they declare that they have no known competing financial interests or personal relationships that could have appeared to influence the work reported in this paper.
